# Evaluation of Growth Performance and Environmental Impact of *Hermetia illucens* Larvae Reared on Coffee Silverskins Enriched with *Schizochytrium limacinum* or *Isochrysis galbana* Microalgae

**DOI:** 10.3390/ani14040609

**Published:** 2024-02-13

**Authors:** Sara Ruschioni, Daniele Duca, Francesca Tulli, Matteo Zarantoniello, Gloriana Cardinaletti, Lorenzo Corsi, Ike Olivotto, Danilo Basili, Simona Naspetti, Cristina Truzzi, Nunzio Isidoro, Paola Riolo

**Affiliations:** 1 Dipartimento di Scienze Agrarie, Alimentari ed Ambientali, Università Politecnica delle Marche, Via Brecce Bianche, 60131 Ancona, Italy; s.ruschioni@staff.univpm.it (S.R.); d.duca@staff.univpm.it (D.D.); lorenzo.corsi1993@gmail.com (L.C.); n.isidoro@staff.univpm.it (N.I.); 2 Dipartimento di Scienze Agro-Alimentari, Ambientali e Animali, Università di Udine, Via Sondrio 2/A, 33100 Udine, Italy; francesca.tulli@uniud.it (F.T.); gloriana.cardinaletti@uniud.it (G.C.); 3 Dipartimento di Scienze della Vita e dell’Ambiente, Università Politecnica delle Marche, Via Brecce Bianche, 60131 Ancona, Italy; m.zarantoniello@staff.univpm.it (M.Z.); i.olivotto@staff.univpm.it (I.O.); c.truzzi@staff.univpm.it (C.T.); 4 Department of Chemistry, University of Cambridge, Lensfield Road, Cambridge CB2 1EW, UK; 5 Dipartimento di Scienze e Ingegneria della Materia, dell’Ambiente ed Urbanistica, Università Politecnica delle Marche, Via Brecce Bianche, 60131 Ancona, Italy; simona@agrecon.univpm.it

**Keywords:** *Hermetia illucens*, growth performance, sustainability, circular economy, coffee silverskin, microalgae, *Schizochytrium limacinum*, *Isochrysis galbana*

## Abstract

**Simple Summary:**

The feeding and nutrition of *Hermetia illucens* becomes an important issue due to its ability to convert organic by-products obtained from agri-food chains into extremely valuable biomass. In this context, coffee silverskin, a by-product of coffee roasting, enriched with 5%, 10%, 20%, and 25% of microalgae (*Schizochytrium limacinum* and *Isochrysis galbana*), was investigated as a basal rearing substrate for *H. illucens*. The study also analyzed the evaluation of the environmental sustainability of diets. The results showed that the inclusion of microalgae in the diets led to an increase in larval growth performance, especially in larvae fed with *S. limacinum*. Conversely, the higher the proportion of microalgae, the greater the environmental impact of larval production. Therefore, considering that the 10% *S. limacinum* inclusion achieved the best waste reduction index and showed only minor differences compared to the higher inclusions of this microalgae, it can be considered a compromise between the nutritional properties of the insects and sustainability. Moreover, considering that the rearing substrate enriched with 10% *S. limacinum* achieved the best waste reduction index and showed only minor differences in terms of nutritional value of *H. illucens* prepupae compared to the higher inclusions of this microalgae, it can be considered a compromise for sustainable insect production.

**Abstract:**

*Hermetia illucens* is a promising insect due to its ability to convert low-value substrates as food chain by-products into highly nutritious feed. Its feeding and nutrition are important issues. The aim of this work was to investigate the effect of different substrates consisting of coffee silverskin, a by-product of the roasting process, enriched with different inclusions of microalgae (5%, 10%, 20%, and 25%), *Schizochytrium limacinum,* and *Isochrysis galbana*, combined with the assessment of environmental sustainability by LCA. In general, the addition of microalgae led to an increase in larval growth performance due to the higher content of protein and lipids, although *S. limacinum* showed the best results with respect to larvae fed with coffee silverskin enriched with *I. galbana*. A higher prepupal weight was observed in larvae fed with 10%, 20%, and 25% *S. limacinum*; shorter development times in larvae fed with 25% of both *S. limacinum* and *I. galbana*; and a higher growth rate in larvae fed with 25% *S. limacinum*. The 10% *S. limacinum* inclusion was only slightly different from the higher inclusions. Furthermore, 10% of *S. limacinum* achieved the best waste reduction index. The greater the inclusion of microalgae, the greater the environmental impact of larval production. Therefore, the addition of 10% *S. limacinum* appears to be the best compromise for larval rearing, especially considering that a higher inclusion of microalgae did not yield additional benefits in terms of the nutritional value of *H. illucens* prepupae.

## 1. Introduction

Coffee is one of the most consumed beverages in the world and the second most important commodity produced by developing countries (after petroleum) [1]. In 2019/2020, about 10 million tons of bagged coffee were consumed worldwide [2], generating a large amount of by-product (pulp, husks, silverskin, and coffee waste) in this industry. In 2022, Italy was the third-largest importer of green coffee in the world (after the USA and Germany) and the second-largest exporter of roasted coffee in the EU (after Germany). Coffee is imported into Italy for roasting, which is the industrial process that determines the final taste and aroma of the cup of coffee. Coffee silverskin (CB), the tegument of the outer layer of the coffee bean, is the only by-product of the roasting process. Since roasting 10 tons of coffee produces about 83 kg of CB [3], this by-product is a potential pollutant if simply discarded into the environment [4]. Coffee silverskin is high in fiber (62%), protein (19%), fat (from 1.6 to 3.3%), and minerals (5% ash). In addition, this by-product has an antioxidant effect due to the presence of melanoidins and phenolic compounds [3,5] (e.g., chlorogenic acid). Various utilization alternatives have been explored to both reduce the environmental impact and increase the added value of CB, such as the recovery of functional ingredients for potential applications in the food, pharmaceutical, and cosmetics industries, direct energy production, composting, bioenergy, and the production of biomaterials [3,6,7,8,9,10]. The conversion of CB by larvae of the black soldier fly (BSF), *Hermetia illucens* (Diptera: Stratiomydae), represents an interesting recycling alternative that complies with the principles of sustainability and the circular economy. Indeed, BSF larvae are able to convert large amounts of organic by-products into valuable nutrients (proteins and lipids) that could be used as ingredients for animal feed [11,12,13,14,15,16,17,18,19,20,21,22,23,24,25] and renewable energy [26,27,28]. In addition, their frass (larval excreta mixed with substrate residues) can be used as an effective organic fertilizer to develop more sustainable agriculture [29].

In 2017, the European Commission authorized the use of insects as an ingredient in aquafeed (Reg. (EU) 2017/893), including BSF. In addition, in 2021, the EU authorized the use of insect proteins in poultry and pig feed (Reg. (EU) 2021/1372). BSF larvae are rich in nutrients such as fat, protein, and high-quality amino acids and minerals, making them a good source of protein. Feed enriched with BSF larvae appears to improve growth performance and digestibility in pigs and poultry compared to other protein feeds. At the same time, BSFL larvae are also rich in minerals and chitin and have antioxidant and positive effects on the immune system [30]. 

The composition of BSF larvae guarantees a supply of proteins and essential amino acids very similar to that of conventional aquafeed protein sources [31,32], but although they have a high fat content, the proportion of polyunsaturated fatty acids is very low [17,23,33,34,35,36,37,38]. However, the nutritional composition of BSF larvae, especially the lipid content and fatty acid profile, can be modulated by the feed substrate according to the nutritional requirements of the fish [23,39,40,41,42,43]. In addition, the quality of feed substrate also influences the performance of BSF larvae and their bioconversion efficiency [12,20,24,44,45,46,47].

To the best of the authors’ knowledge, few studies have been conducted on the environmental impact of BSF larvae production [16,19,48,49,50]. Nowadays, the most digestible and nutritious ingredients for aquafeed are fishmeal and fish oil (FAO, 2020). However, these ingredients are no longer sustainable, and alternatives are needed to promote sustainability while maintaining fish welfare [51]. Feed is the most important production cost in aquaculture [52]. Researchers have examined the environmental sustainability of various alternative ingredients using Life Cycle Assessment (LCA) and reported that insect-derived protein and lipid sources are the most environmentally friendly solutions compared to other alternatives such as microalgae [50]. It should be noted that LCA analyses take into account the most important impact categories, such as global warming, resource depletion, acidification, and eutrophication, but neglect some important aspects, such as the decline of wild fish. With the help of LCA, it is possible to understand whether the proposed alternative useful for replacing fishmeal and fish oil can have a significant impact on other very important issues; otherwise, there is a risk that one problem is solved but many others are created. 

In this context, as part of the project “New nutrients for the production of valuable fish species—Nutrifish”, the locally available by-product of coffee roasting (coffee silverskin) was used as a basal rearing substrate for BSF larvae. This basic substrate for rearing was enriched with an increasing amount of biomass of *Schizochytrium limacinum* or *Isochrysis galbana* microalgae as a source of valuable vitamins, proteins, and polyunsaturated fatty acids [53,54,55,56,57]. In particular, the present study was to investigate: (i) larval growth performance; (ii) final total insect biomass; (iii) macronutrient composition of mature larvae (prepupae); (iv) bioconversion efficiency; and (v) assessment of the environmental sustainability of insect biomass production by LCA.

## 2. Materials and Methods

### 2.1. Ethics 

All procedures involving animals were performed in compliance with the Italian legislation on experimental animals. For invertebrates such as insects, no specific authorization is required under current legislation. 

### 2.2. Diet Preparation

The by-products from the roasting of the coffee blend of *Coffea arabica* and *Coffea canephora*, the coffee silverskin (CB), were collected at Saccaria Caffe S.R.L. (Marina di Montemarciano, Ancona, Italy). The coffee silverskin was packed in an airtight plastic bag, brought to the laboratory, and stored at −20 °C to prevent decomposition. Before use, the CB was brought to room temperature and ground to a particle size of 2.0 ± 0.4 mm using a food mill (Ariete, De’Longhi Appliances Srl, Ancona, Italy). The obtained material was then thoroughly mixed with increasing proportions (5%, 10%, 20%, 25%) of freeze-dried microalgae, *Schizochytrium limacinum* (S) or *Isochrysis galbana* (I) (provided by AlghItaly Società Agricola S.R.L., Sommacampagna, VR, Italy), and demineralized water to produce an experimental diet with 70% moisture for BSF larvae. The feed mixtures CB:S and CB:I were used as experimental diets (n = 8), and CB served as a control (Table 1). The samples of CB, S, and I were stored at −20 °C for further analysis.

### 2.3. Experimental Setup

BSF larvae were provided by Smart Bugs s.s. (Ponzano Veneto (TV), Italy). For each experimental diet, 6 groups of 100 six-day-old larvae were isolated, cleaned from the initial feeding substrate, counted by hand, weighed (RADWAG Wagi Elektroniczne, AS 82/220.X2, Varsaw, Poland), and placed in plastic containers (10 cm × 17.5 cm × 7 cm) [17] together with the rearing substrate (70 g/replicate, n = 6). Each week, feed substrate was added until 40% of the prepupae had emerged, using a feed rate of 100 mg/day/larva [11]. The previously weighted containers were shielded with fine-mesh cotton gauze (40 × 30 cm), covered with a lid, and, in view of the high migratory tendency of the mature larvae, additionally wrapped with organza. The lid was provided with a single ventilation hole (4.5 cm Ø) [14]. Each container was inspected daily, and the feeding substrate was rearranged as necessary to promote aeration and prevent quality degradation. The larvae were kept under constant conditions in a climate chamber (T: 27 ± 1 °C, RH: 65 ± 5%, 0:24, L:D photoperiod) [14]. As soon as 40% of prepupae appeared in each replicate, the prepupae and mature larvae were manually isolated from the feed with tweezers and a brush, cleaned of substrate residues, washed in water, dried on a piece of paper, and counted. The prepupae were identified by the change in the color of the integument from larval white to black [58]. The total final insect biomass and the remaining rearing substrate (larval excreta mixed with substrate residues) were weighed. The final biomass was stored at −20 °C for further analysis. 

### 2.4. Growth Performance and Conversion Efficiency of the BSF Larvae

The larval development time was calculated as the number of days between the start of the experiment and the observation of 40% of the prepupa in each replicate.

The larval survival rate was calculated as follows: Survival Rate (%) = number of surviving larvae/number of initial larvae × 100.

The growth of the larvae was measured on the basis of the biomass obtained and expressed as growth rate (GR) using the following formula [59], with a higher GR indicating faster larval growth:

Growth rate (g/day) = (final weight (g) − initial weight (g))/time needed to reach prepupal phase (d)(1)

The ability of larvae to reduce food was calculated by the waste reduction index (WRI) according to the following formula, with high WRI values indicating good food reduction efficiency [17]:

WRI = [(total feed (g) − residue(g)) /total feed (g)]/feeding time (d) × 100(2)

Feed conversion, i.e., the amount (kg) of feed required to achieve a weight gain of one kg, was calculated as the feed conversion ratio (FCR) according to the following formula [12], with low FCR values indicating high feed conversion.

FCR = (distributed substrate (g) − residual substrate (g))/(total final biomass (g) − total initial biomass (g))(3)

All parameters were calculated on the basis of fresh mass [12,13,17].

### 2.5. Diet Ingredients and Larval Composition Analysis

The freeze-dried biomasses of coffee silverskin, *S. limacinum,* and *I. galbana*, insect diets, and insects were analyzed in the laboratories of the University of Udine (Udine, Italy) for moisture (Method #950.46), crude protein, CP as Kjeldhal nitrogen (Method #976.05), and ash (Method #920.153) according to AOAC (2006), and for total lipid content according to 54. The kP factor of 4.67 was applied to Kjeldhal N to estimate the crude protein content of BSF larvae, according to Janssen et al. [60].

### 2.6. Life Cycle Assessment

In order to support the choice of the most sustainable experimental diet for feeding larvae with roasted coffee by-products enriched with two different microalgae species in the industrial rearing of BSF, a simplified life cycle assessment (LCA) of insect biomass production was performed to obtain a first indication of the least impacting option from an environmental point of view. For this purpose, the LCA method was carried out according to the requirements of the ISO 14040 [61] and 14044 [62] standards. We included microalgae production and the rearing process based on primary data collected directly during the study to get an idea of the impact of BSF production, using the same approach as many other LCA analyses, which facilitates the comparison of results.

#### 2.6.1. Aim and Scope

**Figure 1:**
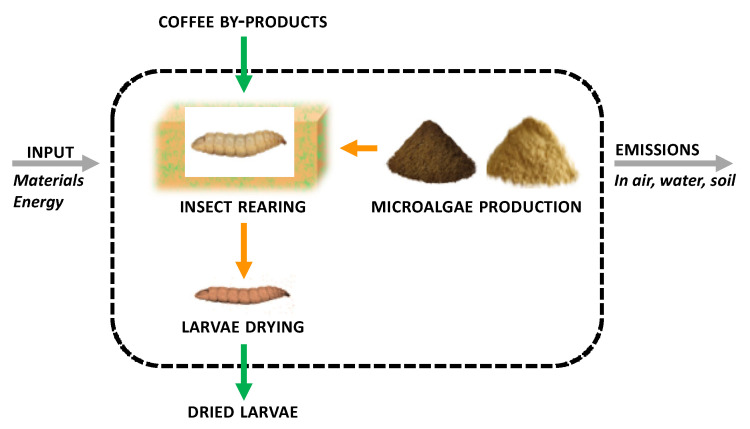
System boundaries for the life cycle assessment (LCA) that were considered in the study.

The aim was to quantify the environmental impact of BSF insect biomass production based on four (5%, 10%, 20%, and 25%) freeze-dried microalgae inclusions in the basal rearing substrate, a by-product of the roasted coffee industry (coffee silverskin-CB). The alternative functional units of 1 kg of protein and 1 kg of lipid were performed as described by other authors [19], and the protein and lipid content of the final BSF biomass reared on the different diets was evaluated as described in Section 2.5. The functional unit was 1 kg of freeze-dried insect biomass produced, in accordance with the scientific literature, to facilitate the comparison of results. The system boundaries of the analysis are shown in Figure 1.

For the production of microalgae, primary data were provided by an Italian producer, while for the production of insects, the results of experiments conducted and previously reported by entomologists from the Dipartimento di Scienze Agrarie, Alimentari ed Ambientali–Università Politecnica delle Marche were used, integrated with secondary data from the literature [16,48,49,63,64,65] and internationally recognized LCA databases (Ecoinvent v. 3.5, Agri-footprint v. 4.0). The photobioreactors used for the production of microalgae are horizontal tubes equipped with storage tanks, pumps, and a system to prevent overheating.

#### 2.6.2. Life Cycle Inventory

For microalgae production, on-site investigations were carried out to collect all data on the photobioreactor-based production process and the associated inputs, outputs, and emissions. It was not possible to obtain specific data for the two different microalgal species, but average values were collected. In detail, the following information was collected through specific questionnaires as primary data: water, chemical and energy consumption, and wastewater produced. These primary data cannot be reported for confidentiality reasons.

For the production of insect biomass, the data were collected at the Dipartimento di Scienze Agrarie, Alimentari ed Ambientali, Università Politecnica delle Marche–Entomology Laboratories. The primary data refer to the feed ingredients used (amount of microalgae and coffee silverskins used for the formulation of each feed), to the relative output, including the amount of insect biomass produced and residual rearing substrate (excrements of larvae mixed with substrate residues and exuviae), and to the corresponding FCR values. Secondary data were used for water and energy consumption and direct greenhouse gas emissions [49,64].

#### 2.6.3. Life Cycle Impact Assessment

The LCA results were calculated using the SimaPro 9 software application (PRé Sustainability B.V., Amersfoort, The Netherlands, 2021). In line with other similar studies, the ReCiPé 2016 life cycle assessment method was used. The following impact categories were taken into account: global warming, freshwater eutrophication, water use, land use/conversion, mineral resources (materials), and fossil resources (energy).

### 2.7. Statistical Analysis

The statistical analyses were performed with the statistical program R. Data on prepupal weight, development time, survival rate, growth rate (GR), waste reduction index (WRI), feed conversion ratio (FCR), and chemical composition of BSF larvae fed the eight different experimental diets and the control diet (Table 1) were compared using the Kruskal–Wallis test or one-way analysis of variance (ANOVA), depending on the data distribution. The pairwise Wilcoxon post hoc test, or Tukey–Kramer’s Honestly Significant Difference (HSD) multiple comparison post hoc test, was used for the mean separation (*p* < 0.05) between the diets tested. The normality of the data was assessed using the Shapiro–Wilk test, and multiple testing corrections were performed using the Benjamini–Hochberg (BH) method. 

## 3. Results

### 3.1. Growth Performance and Conversion Efficiency of BSF Larvae

The effects of the different feeds tested on the growth performance and quality characteristics of BSF are shown in Table 2 and Table 3.

It was found that all experimental diets significantly increased the weight of the prepupae, the development time, and the biomass obtained compared to the control. With the same proportion of basal substrate (CB), larvae enriched with *S. limacinum* performed better than larvae fed with *I. galbana* in terms of prepupal weight, biomass gained, and ability to reduce the feeding substrate. In addition, the survival rate of BSF larvae was not affected by feeding.

In particular, the growth dynamics of BSF larvae fed with the different tested diets were investigated by considering prepupal weight, survival rate, development time, and growth rate (Table 2). Differences were observed in the diets enriched with *S. limacinum* compared to the control group in prepupal weight (Table 2), which increased in a dose-dependent manner (the higher the microalgae content, the higher the insect weight), with the weight of larvae fed with 25S being higher (df = 4, *p* = 0.0001). In the diets enriched with *I. galbana*, the prepupal weight of larvae fed all diets increased compared to the control, but 10I and 25I showed the highest prepupal weight (df = 4, *p* = 5.75 × 10^−5^). When comparing all experimental diets formulated with different amounts of both microalgae species, the authors found that *S. limacinum* supported insect growth performance better than *I. galbana*, with emphasis on prepupal weight, especially in larvae fed diets 20S and 25S (df = 8, *p* = 2 × 10^−16^).

The percentage of microalgae in diets significantly influenced the larval development time (Table 2). More specifically, the larvae fed with 25S and 25I diets showed a shorter development time than those fed with the other diets tested (df = 4, *p* = 7.82 × 10^−6^).

The different concentrations of the two microalgae significantly influenced the growth rate (GR), indicating a high efficiency of larval growth (Table 2). Larvae fed with 25% *S. limacinum* (25S) showed higher GR values compared to the other tested diets (df = 4, *p* = 3.65 × 10^−5^). Among the *I. galbana*-enriched diets, larvae fed 10I and 25I showed higher GR values (df = 4, *p* = 5.92 × 10^−5^).

Both the WRI and FCR of BSF larvae were affected by the different diets tested. Larvae fed with C had higher FCR values (df = 8, *p* = 9.41 × 10^−7^) and lower WRI values (df = 8, *p* = 7.08 × 10^−8^) (Table 3). Larvae fed with 10% *S. limacinum* (10S) had statistically higher WRI values compared to larvae fed with all other tested diets (df = 4, *p* = 3.32 × 10^−5^). Within the *I. galbana*-fed larvae group, 20I had higher WRI values (df = 4, *p* = 0.0002). In terms of FCR, we found that 5S, 20S, 25S, 10I, and 25I caused significantly lower values of FCR than all other diets tested (df = 4, *p* = 0.003).

### 3.2. Diet Ingredient and Larval Composition Analysis

#### 3.2.1. Diet Composition Analysis

The chemical composition of the experimental diets with coffee silverskin and increasing (5%, 10%, 20%, and 25%) content of *S. limacinum* and *I*. *galbana* is shown in Table 4.

Increasing the addition of *S. limacinum* dry biomass to the coffee silverskin basal diet resulted in an increased protein content, ranging from 20.96 g/100 g dry matter of the control diet to 31.07 g/100 g dry matter of the 25S diet. The fat content of the feed was limited as it was influenced by the addition of *S. limacium*.

The addition of increasing amounts of *I. galbana* biomass to the coffee by-products as a substrate for the growth of *H. illucens* resulted in an increase in substrate protein content and a significant increase in total fat content up to 3.56 g/100 g, which corresponds to an addition of 25% (diet 25I) compared to the control diet. 

#### 3.2.2. Insect Composition Analysis

The characterization of the proximal composition of the BSF larvae reared on the different experimental diets is shown in Table 5.

The addition of both microalgae to the basic substrate for rearing (coffee silverskin) led to a significant modulation of the nutrient composition of the BSF larvae.

Compared to the composition of the experimental diets, larvae of BSF reared on diets containing increasing amounts of *S. limacinum* had proportionally higher nutritional value in terms of both protein and lipid content, confirming the initial hypothesis that modulation of substrate composition also affects the nutritional composition of BSF. The highest protein content was found in larvae reared on the 25S diet (18.3% CP). On the other hand, the lipid content of the larvae was significantly affected by the increasing content of microalgae up to diet 20S. A content of *S. limacinum* of more than 20% did not significantly change the lipid content of the larvae (*p* > 0.05).

The proximate composition of BSF larvae reared on diets containing increasing amounts of *I. galbana* resulted in a significant increase in both protein and lipid content compared to larvae fed the control diet (*p* < 0.05). However, there is no corresponding change in protein content in larvae proportional to increasing proportions of microalgal biomass greater than 10%; the highest lipid contents in larvae were found at 25% *I*. *galbana* (4.95%).

### 3.3. Life Cycle Assessment

The LCA analysis yielded the results listed in Table 6 and Table 7 for the feeds containing *S. limacinum* and *I. galbana*, respectively. All values refer to the same functional unit, namely 1 kg of dried larvae produced. Consequently, the values can be directly compared with each other. The variability of the average values was always less than 10%. 

The results show that all environmental impacts considered are strongly influenced by the percentage of microalgae in the diet. The coffee silverskin is a remnant and is not related to the impact of production. As a result, the lowest impacts were calculated for feeds 5I and 5S. The contribution of the larval drying process is not relevant compared to microalgae production, especially at a level of 10% or more in the diet, where the impact of microalgae accounts for more than 80% of the total impact. The impact of microalgal production is mainly due to the high energy input and the low maturity of the technology.

The leftover insect substrate was not considered waste to be disposed of, as it could be used in sustainable agriculture as a fertilizer to partially replace the chemical fertilizer and provide several benefits in terms of promoting plant growth and increasing tolerance to abiotic stress and resistance to pathogens and pests [29]. However, the actual impact of this specific substitution in terms of the impact categories considered is difficult to estimate [66] and, in this case, limited compared to the results obtained (e.g., up to a saving of 2 kg CO_2_ eq/kg of dried larvae produced for global warming), mainly due to the low nitrogen content of the remaining rearing substrate (about 4% on a dry matter basis). For these reasons, this limited and uncertain impact reduction was not included in the calculation.

As an additional reference, the results were also expressed using the alternative functional units related to 1 kg protein (Table 8 and Table 9) and 1 kg lipid, based on the different protein and lipid contents (Table 10 and Table 11) of the BSF larvae reared on the different diets listed in Table 5. 

The increase in protein and lipid content of BSF larvae fed the experimental diets with the highest microalgae content partially offset the increase in effects, but only to a very limited extent.

## 4. Discussion

Several studies have been conducted on the nutritional requirements of BSF [12,14,15,40] and on the evaluation of by-products used as feed [15,17,67,68], but much more information is needed. For this reason, the growth performance of BSF and its impact on the environment were investigated when reared on potentially environmentally acceptable feed substrates, *S. limacinum* or *I. galbana*-enriched coffee silverskin. The use of a residual substrate has, in general terms, the potential to improve the sustainability of a product by substituting a dedicated input, but this sustainability has to be proven with studies and results. For this reason, we carried out an LCA supporting the insect production results. This study provided data on the effects of enriching the by-product coffee silverskin with different microalgae inclusions (*S. limacinum* and *I. galbana*) on the growth performance of BSF. These rearing substrates were characterized by a different chemical composition, which influenced the growth performance of the larvae. Considering that BSF stores most of their nutrients during larval development, as the adult stage is unable to feed [69], it is clear that the quality of the rearing substrate is crucial for their fitness [45,70,71]. Therefore, the nutrient composition of the rearing substrates had a major impact on critical production factors such as survival rate and larval weight, which are positively correlated with growth rate [12,67,72,73]. As nutrition is a crucial factor in insect fitness and environmental impact, studying its effects on insects, processing conditions, and final product characteristics is very important [16]. In terms of producing more sustainable ingredients for aquafeed, it is of great interest to influence the nutritional value of insects by enriching the rearing substrate. Cb in particular consists mainly of fibers and carbohydrates, which makes this by-product unsuitable for the formulation of fish feed [74]. Fish feed must generally have a high protein and lipid content, followed by very low amounts of fiber and carbohydrates, which are mainly indigestible for fish [75]. Furthermore, the use of microalgae as a component of aquafeed has been extensively studied because of their beneficial properties, but they may only be used in small quantities for economic reasons and because they can lead to an impairment of nutrient absorption in the fish gut [76]. On the contrary, various studies have shown that the use of insects grown on a substrate enriched with microalgae can positively transfer important bioactive molecules to the fish feed and thus have a positive effect on the overall health of the fish [77,78].

In this study, in agreement with Truzzi et al. [57], both microalgae species showed a higher crude protein (average of 16.40 g/100 g dry matter for both microalgae) and lipid content (average of 6.15 g/100 g dry matter for *S. limacinum* and an average of 4.26 g/100 g dry matter for *I. galbana*) than the coffee silverskin (protein 13.47 g/100 g dry matter, lipids 3.18 g/100 g dry matter). Consequently, the best growth performance was achieved with the higher microalgae contents. Although both experimental diets significantly increased larval weight and survival, *S. limacinum* provided a greater benefit than *I. galbana*. As the composition of the two microalgae was different, *S. limacinum* provided a higher amount of proteins and lipids compared to *I. galbana*, with an emphasis on unsaturated fatty acids.

A shorter larval development time was observed in BSF larvae reared on microalgae-enriched diets compared to the control diet (19 days on average for both microalgae versus 39 days for CB). The development time was also shorter compared to the reports of other authors [12,45,46,79]. The development time of larvae reared with higher microalgal inclusions (25S and 25I) was 18 days, similar to Barragan-Fonseca et al. [80]. The development time of larvae reared only with coffee silverskin was very long, showing that rearing with low-fat substrates tends to negatively affect larval fitness. This result confirms what is reported in the current literature, namely that BSF larvae require a high-fat diet to store the body fat needed for development [69,70,81]. If the rearing substrate has a low lipid content, the larvae take longer to acquire their body fat and complete their development [45].

Substrates enriched with microalgae proved to be more effective compared to coffee silverskin, also in terms of increasing prepupal weight (average 164.6 mg for *S. limacinum*, average 123.9 mg for *I. galbana,* and average 72.4 mg for CB). Indeed, the weight of larvae reared on the experimental diets (0.1363 g on average) was significantly higher than that of larvae reared on the control diet (0.0724 g), and they were comparable to the values reported in the literature by different authors [45,70,81,82,83]. In agreement with Nguyen et al. [45,81], we observed that larval weight increased with both the protein and fat content of the diet.

Faster larval development indicates a higher growth rate in relation to the final biomass obtained in a shorter time, which is an advantage for rearing [84]. In fact, the growth rate was also better for larvae reared on microalgae-enriched feed. In particular, the highest growth rate was recorded for the diet enriched with *S. limacinum*, followed by the diet enriched with *I. galbana*, while the larvae reared only with coffee silverskin showed the lowest growth rate. These results confirm that although BSF require proteins and lipids in their diet, an excessive amount of these macromolecules in the substrate (e.g., lipids in an *I. galbana*-enriched diet) is often detrimental to the growth rate, as the larvae may have problems metabolizing too much fat during the metamorphosis process [45,85]. The survival rate obtained in the present study for all experimental and control diets was in line with the range reported by several authors for a variety of rearing substrates [12,19,79,81,83].

Nowadays, by-products are becoming increasingly important feed ingredients [86], especially as they are considered less economically valuable and less polluting than the main product [87]. Their advantage also depends on how well these by-products can be converted into body mass by the insects [12]. For this reason, the combination of different substances, including by-products, can be used as an efficient feed, and their composition is the most important variable to determine the FCR [88]. Basically, FCR is the amount of feed needed to achieve a weight gain of one kilogram for the farm animal. In order to achieve better FCRs that provide optimal economic and environmental benefits when using BSF as an alternative feed, it is necessary that the tested feeds are directly and effectively utilized by the insects. In this work, FCR was more favorable for microalgae-enriched feeds, indicating better utilization of feed nutrients. The FCR values obtained in this work (from 4.5 to 9.5 for microalgae-enriched feeds; 34.2 for control feeds) were much higher than the FCR values obtained by Oonicx et al. [12] (from 1.4 to 2.6), who studied by-products from food production. These values were lower than those obtained by Rehman et al. [89] (from 6.3 to 10.1), who studied larvae reared on dairy fertilizer and soybean residues. The results of this work show that high-protein diets lead to lower FCRs, as also reported by Oonincx et al. [12]. Indeed, the composition and density of proteins are important for insects, as they do not require energy to maintain a constant body temperature [90,91]. 

Finally, the waste reduction index was also analyzed, a parameter used to calculate the insect’s ability to reduce waste and the conversion efficiency of food into biomass. The higher the WRI value, the more effective the larvae are at converting by-products [19]. In this work, the WRI value of 10S (2.6%/day) was found to be higher than the other diets in the experiment (less than 2.2%/day). This WRI value observed in this work agrees with that of Leong et al. [92], who reared BSF larvae on fruit waste from a cafeteria (2.8%/day) and palm decanter cake (2.8%/day), but much less than Bava et al. [19], who reared larvae on okara (4.9%/day), corn distillers (3.2%/day), chicken feed, and brewers grain feed (3.0%/day). 

The addition of microalgal biomass to the rearing substrate led to a significant modulation of the nutrient composition of the BSF larvae. The proximate composition of BSF larvae reared on diets containing increasing levels of microalgae resulted in a significant increase in both protein and lipid content compared to larvae reared on coffee silverskin alone, confirming the hypothesis that modulation of substrate composition also affects the nutritional composition of insect larvae [23,39,40,41,42]. In particular, BSF larvae reared on diets containing increasing levels of *S. limacinum* had proportionally higher nutritional value, both in terms of protein and fat content.

The protein content of the larvae was significantly influenced by the increasing proportion of microalgae and reached the highest content in larvae reared on the 25S and 25I diets (18.3% CP). This trend was linear with the increase in protein content in the proximate composition of the diet. The protein content determined in the present study was lower than that reported by several authors [19,34,93], but in these papers, the BSF larvae were reared on much higher protein diets, which may have influenced the nutritional composition of the larvae.

A different trend was observed in the lipid content of the larvae, which changed with increasing levels of *S. limacinum*, with 20I and 25I having the highest levels (7.74 and 7.93 g/100 g, respectively) and were not influenced by increasing levels of *I. galbana*. Although the diets containing *I. galbana* had a significantly higher lipid content than the diets containing *S. limacinum*, the larvae had similar lipid levels in their biochemical composition to the larvae reared on 5S and 10S diets.

The results of the LCA show that the calculated environmental impacts are high compared to the values given in the literature for BSF production based on various substrates [48] for all impact categories considered. This is mainly due to the high impact of microalgae production and is in line with the results of other studies [94,95]. In one research study, microalgae were considered a less environmentally friendly substitute for insect meals [49]. Therefore, it is very likely that the use of microalgae for insect production increases the impact of insect production. In order to properly evaluate the different solutions, the nutritional quality aspects should be considered at the same time, as reported in the present study. It should also be emphasized that the impact of fishmeal is in some cases higher than that of 5S and 5I86 and that the negative impact of fishmeal on the decline of marine fish stocks is not considered in the LCAs.

To summarize, the data available in the literature do not allow us to say which is the best growth substrate for BSF larvae. Therefore, the results of this work can be considered an enrichment of the database, collecting information on how the performance of BSF larvae can be influenced by the substrates. Also, in terms of LCA, it might be useful to limit the microalgae content or to choose a more sustainable microalgae production. The latter will probably be possible in the future with the improvement of microalgae production technology. It should be noted that in certain scenarios where the side streams are also recycled, such as spent substrate as compost, or where insect production is considered as an alternative waste disposal option, a significant reduction in impact could be achieved. 

Although this study was conducted under laboratory conditions, it provides a good perspective on the use of BSF as a by-product degrader.

## 5. Conclusions

As part of the Nutrifish project, authors analyzed the influence of these microalgae-enriched substrates on the fatty acid profile of BSF prepupae [57] and the occurrence of antibiotic resistance genes in this BSF larvae rearing chain [96]. In addition, BSF was reared on these diets to study its use as a sustainable terrestrial ingredient for aquafeed production [25,43] and to investigate the physiological and behavioral responses of zebrafish to insect-based diets [77]. Last but not least, this work evaluates the growth performance of BSF and their impact on the environment when reared on potentially environmentally benign feed substrates (microalgae-enriched coffee silverskin) in order to propose them as an alternative feed ingredient in aquaculture. This study is important because several studies on the rearing of BSF and the evaluation of by-products used as feed have been conducted to date [15,17,69,70], but there is still little information on BSF. Considering the amount of coffee produced and therefore the amount of its by-products, as well as the pollution caused by coffee mites that are simply discarded into the environment, the need for different utilization alternatives to both reduce the environmental impact and increase the added value of CB is obvious.

The choice of rearing substrate for BSF is very important for growth performance and for an environmentally friendly product. In this work, it was confirmed that the by-product coffee silverskin, which is a major environmental hazard when used as a feed additive to be upgraded, contains low amounts of proteins and lipids to ensure good growth performance of BSF. However, by enriching this substrate with *S. limacinum* and *I. galbana*, two ingredients that contain the right amount of nutrients, these problems could be solved. Of the two microalgae, *S. limacinum* proved to be more beneficial than *I. galbana*.

Considering that the rearing substrate for BSF also contributes significantly to the environmental impact of larval production, as shown by the LCA results, it is very important to study it in depth, from the sustainability of the substrate components to its effect on the insects. This is necessary to better evaluate the ecological benefits of insects as alternative feed ingredients. As the sustainability of current *S. limacinum* and *I. galbana* production is limited, the percentage of substrate components should be limited. Moreover, a study proposed the inclusion of spirulina in the coffee-silverskin to enrich BSF final biomass in terms of nutritional value as an alternative aquafeed ingredient in commercial trout production [78]. Further investigations are currently being performed to use agri-food leftovers (tomato, spinach, chickpeas, peas, and wheat), focusing on their availability in a defined geographical area (Regione Marche, Italy), as rearing substrates for BSF larvae to use as non-ruminant livestock feed. Based on the present findings, further research is needed to scale up BSF larvae production and assess the environmental impacts and sustainability of large-scale farming of BSF.

## Figures and Tables

**Table 1:** Experimental diet compositions based on coffee by-product (CB) and different levels of *Schizochytrium limacinum* (S) or *Isochrysis galbana* (I).

Diets	CB (%)	*Schizochytrium limacinum* (%)	*Isochrysis galbana* (%)
5S	95	5	-
10S	90	10	-
20S	80	20	-
25S	75	25	-
5I	95	-	5
10I	90	-	10
20I	80	-	20
25I	75	-	25
C	100	-	-

Experimental diet abbreviations: 5S = 95% CB + 5% S; 10S = 90% CB + 10% S; 20S = 80% CB + 20% S; 25S = 75% CB + 25% S; 5I = 95% CB + 5% I; 10I = 90% CB + 10% I; 20I = 80% CB + 20% I; 25I = 75% CB + 25% I; C = 100% CB.

**Table 2:** Growth performance of BSF larvae reared on different experimental diets consisting of coffee by-products (CB) enriched with *Schizochytrium limacinum* (S) and *Isochrysis galbana* (I) dry biomass and the control (C) (mean ± SD; n = 6).

Diets	Prepupal Weight (mg)	Survival Rate (%)	Development Time (days)	Growth Rate (mg/day)
5S	143.0 ± 12.4 ^cB^	88.2 ± 6.1 ^aA^	20 ± 0 ^cC^	7.0 ± 0.6 ^cC^
10S	161.3 ± 10.7 ^bcAB^	91.0 ± 5.4 ^aA^	19 ± 0 ^bB^	8.4 ± 0.6 ^bB^
20S	173.9 ± 9.6 ^abA^	87.8 ± 3.1 ^aA^	19 ± 0 ^bB^	9.0 ± 0.5 ^bB^
25S	180.2 ± 11.5 ^aA^	84.5 ± 12.6 ^aA^	18 ± 0 ^aA^	9.9 ± 0.7 ^aA^
5I	107.6 ± 5.1 ^d^^**B**^	84.3 ± 3.1 ^a^^**A**^	20 ± 0 ^c^^**C**^	5.3 ± 0.3 ^e^^**C**^
10I	143.1 ± 5.2 ^c^^**A**^	82.0 ± 7.2 ^a^^**A**^	20 ± 0 ^c^^**C**^	7.0 ± 0.3 ^c^^**A**^
20I	113.1 ± 11.8 ^d^^**B**^	85.2 ± 5.6 ^a^^**A**^	19 ± 0 ^b^^**B**^	5.8 ± 0.6 ^d^^**B**^
25I	132.3 ± 11.2 ^c^^**A**^	84.3 ± 6.5 ^a^^**A**^	18 ± 0 ^a^^**A**^	7.2 ± 0.6 ^c^^**A**^
C	72.4 ± 7.7 ^eC^^**C**^	81.0 ± 8.8 ^aA^^**A**^	39 ± 0 ^dD^^**D**^	1.8 ± 0.2 ^fD^^**D**^

Different superscript letters in a column indicate significant differences (*p* < 0.05): ^a,b,c,d,e,f^ small letters indicate a significant difference among all diets; ^A,B,C,D,E^ capital letters indicate a significant difference among diets consisting of coffee by-products (CB) containing different percentages of *S. limacinum* (S) and the control diet (C); **^A,B,C,D^** capital and bold letters indicate a significant difference among diets consisting of coffee by-products (CB) enriched with different percentages of *I. galbana* (I) and the control diet (C). Abbreviations of the experimental diet: 5S = 95% CB + 5% S; 10S = 90% CB + 10% S; 20S = 80% CB + 20% S; 25S = 75% CB + 25% S; 5I = 95% CB + 5% I; 10I = 90% CB + 10% I; 20I = 80% CB + 20% I; 25I = 75% CB + 25% I; C = 100% CB.

**Table 3:** Waste reduction index (WRI) and feed conversion ratio (FCR) of BSF larvae reared on different experimental diets consisting of coffee by-products (CB) enriched with different percentages of *Schizochytrium limacinum* (S) and *Isochrysis galbana* (I) microalgae and control diet (C) (mean ± SD; n = 6).

Diets	WRI (%/day)	FCR
5S	1.5 ± 0.1 ^dC^	5.2 ± 0.3 ^aA^
10S	2.6 ± 0.2 ^aA^	7.21 ± 0.7 ^cB^
20S	2.1 ± 0.3 ^bcB^	5.6 ± 0.8 ^abA^
25S	2.0 ± 0.2 ^cB^	5.0 ± 0.3 ^aA^
5I	1.2 ± 0.1 ^f^^**CD**^	5.8 ± 0.3 ^b^^**B**^
10I	1.4 ± 0.1 ^e^^**B**^	5.1 ± 0.5 ^a^^**A**^
20I	2.2 ± 0.1 ^b^^**A**^	9.5 ± 0.9 ^d^^**C**^
25I	1.3 ± 0.1 ^ef^^**BC**^	4.5 ± 0.5 ^a^^**A**^
C	1.2 ± 0.1 ^gD^^**D**^	34.2 ± 6.4 ^eC^^**D**^

Different superscript letters in a column indicate significant differences (*p* < 0.05): ^a,b,c,d,e,f,g^ small letters indicate a significant difference among all diets; ^A,B,C,D,E^ capital letters indicate a significant difference among diets consisting of coffee by-products (CB) containing different percentages of *S. limacinum* (S) and the control diet (C); **^A,B,C,D^** capital and bold letters indicate a significant difference among diets consisting of coffee by-products (CB) enriched with different percentages of *I. galbana* (I) and the control diet (C). Abbreviations of the experimental diet: 5S = 95% CB + 5% S; 10S = 90% CB + 10% S; 20S = 80% CB + 20% S; 25S = 75% CB + 25% S; 5I = 95% CB + 5% I; 10I = 90% CB + 10% I; 20I = 80% CB + 20% I; 25I = 75% CB + 25% I; C = 100% CB.

**Table 4:** Water (g/100 g), protein, lipid, and ash (g/100 g dry matter) content of the different experimental diets consisting of coffee by-products (CB) enriched with *Schizochytrium limacinum* (S) and *Isochrysis galbana* (I) dry biomass, and the control (C) (mean ± SD; n = 6).

Diet	Crude Protein	Lipid	Ash
5S	21.42 ± 1.10	0.75 ± 0.06	8.62 ± 0.13
10S	22.42 ± 3.34	0.81 ± 0.04	8.71 ± 0.05
20S	28.06 ± 1.15	0.82 ± 0.05	8.84 ± 0.01
25S	31.07 ± 2.19	0.93 ± 0.03	8.91 ± 0.75
5I	23.52 ± 2.26	1.66 ± 0.10	8.80 ± 0.25
10I	24.79 ± 3.18	2.11 ± 0.06	8.65 ± 0.16
20I	25.63 ± 2.41	2.67 ± 0.10	9.22 ± 0.10
25I	27.00 ± 3.22	3.56 ± 0.05	9.79 ± 0.48
C	20.96 ± 1.32	0.71 ± 0.07	8.71 ± 0.22

Abbreviations of the experimental diet: 5S = 95% CB + 5% S; 10S = 90% CB + 10% S; 20S = 80% CB + 20% S; 25S = 75% CB + 25% S; 5I = 95% CB + 5% I; 10I = 90% CB + 10% I; 20I = 80% CB + 20% I; 25I = 75% CB + 25% I; C = 100% CB.

**Table 5:** Chemical composition (g/100 g) of BSF larvae reared on the different experimental diets consisting of coffee by-products (CB) enriched with *Schizochytrium limacinum* (S) and *Isochrysis galbana* (I) dry biomass and the control (C) (mean ± SD; n = 6).

Diet	Crude Protein	Lipid	Ash
5S	14.86 ± 0.42 ^cDC^	4.05 ± 0.14 ^cC^	7.13 ± 0.13 ^abB^
10S	15.59 ± 0.36 ^bCB^	4.88 ± 0.11 ^bB^	6.34 ± 0.16 ^cbC^
20S	16.85 ± 0.21 ^bBA^	7.74 ± 0.12 ^aA^	4.70 ± 0.26 ^deD^
25S	18.29 ± 0.23 ^aA^	7.93 ± 0.35 ^aA^	4.39 ± 0.13 ^eD^
5I	14.86 ± 0.28 ^c^^**BC**^	4.28 ± 0.26 ^cb^^**B**^	7.68 ± 0.01 ^ab^^**A**^
10I	15.59 ± 0.30 ^b^^**AB**^	4.00 ± 0.06 ^c^^**B**^	6.99 ± 0.77 ^abc^^**AB**^
20I	16.85 ± 0.39 ^b^^**A**^	3.81 ± 0.03 ^cd^^**B**^	5.81 ± 0.29 ^cd^^**CD**^
25I	18.29 ± 0.22 ^a^^**A**^	4.95 ± 0.07 ^b^^**A**^	4.35 ± 0.44 ^e^^**D**^
C	13.47 ± 0.53 ^dD^	3.18 ± 0.08 ^dD^	8.12 ± 0.20 ^aA^

Different superscript letters in a column indicate significant differences (*p* ≤ 0.05): ^a,b,c,d,e^ small letters indicate a significant difference among all diets; ^A,B,C,D^ capital letters indicate a significant difference among diets consisting of coffee by-products (CB) enriched with different percentages of *S. limacinum* (S) and the control diet (C); **^A,B,C,D^** capital and bold letters indicate a significant difference among diets consisting of coffee by-products (CB) enriched with different percentages of *I. galbana* (I) and the control diet (C). Abbreviation of the experimental diet: 5S = 95% CB + 5% S; 10S = 90% CB + 10% S; 20S = 80% CB + 20% S; 25S = 75% CB + 25% S; 5I = 95% CB + 5% I; 10I = 90% CB + 10% I; 20I = 80% CB + 20% I; 25I = 75% CB + 25% I; C = 100% CB.

**Table 6:** Environmental impact of the production of 1 kg of dried BSF larvae fed with different experimental diets of coffee by-products enriched with different percentages (5%, 10%, 20%, and 25%) of *Schizochytrium limacinum*.

Impact Category	Unit	5S	10S	20S	25S
Global warming	kg CO_2_ eq	8.0 × 10^3^ + 00	1.8 × 10^5^ + 01	2.7 × 10^3^ + 01	3.1 × 10^1^ + 01
Freshwater eutrophication	kg P eq	3.7 × 10^4^ − 03	7.9 × 10^6^ − 03	1.1 × 10^5^ − 02	1.3 × 10 − 02
Land use	m^2^a crop eq	4.0 + 10^9^ − 01	8.8 × 10^9^ − 01	1.2 × 10^9^ + 00	1.4 × 10^6^ + 00
Mineral resource scarcity	kg Cu eq	2.7 × 10^2^ − 02	6.5 × 10^8^ − 02	9.8 × 10^3^ − 02	1.1 × 10^2^ − 01
Fossil resource scarcity	kg oil eq	1.9 × 10^7^ + 00	4.6 × 10^3^ + 00	6.8 × 10^7^ + 00	7.8 × 10^3^ + 00
Water consumption	m^3^	1.7 × 10^5^ − 01	4.0 × 10^5^ − 01	5.9 × 10^8^ − 01	6.8 × 10 − 01

**Table 7:** Environmental impact of the production of 1 kg of dried BSF larvae fed with different experimental diets composed of coffee by-products enriched with different percentages (5%, 10%, 20%, and 25%) of *Isochrysis galbana*.

Impact Category	Unit	5I	10I	20I	25I
Global warming	kg CO_2_ eq	8.7 × 10^8^ + 00	1.3 × 10^7^ + 01	4.4 × 10^8^ + 01	2.7 × 10^7^ + 01
Freshwater eutrophication	kg P eq	4.0 × 10^4^ − 03	6.0 × 10 − 03	1.8 × 10^5^ − 02	1.1 × 10^6^ − 02
Land use	m^2^a crop eq	4.4 × 10^3^ − 01	6.6 × 10^6^ − 01	2.0 × 10^9^ + 00	1.3 × 10^1^ + 00
Mineral resource scarcity	kg Cu eq	2.9 × 10^9^ − 02	4.7 × 10^9^ − 02	1.6 × 10^3^ − 01	9.9 × 10^7^ − 02
Fossil resource scarcity	kg oil eq	2.1 × 10^6^ + 00	3.3 × 10^9^ + 00	1.1 × 10^3^ + 01	6.9 × 10^7^ + 00
Water consumption	m^3^	1.9 × 10^2^ − 01	2.9 × 10^9^ − 01	9.8 × 10 − 01	6.0 × 10^6^ − 01

**Table 8:** Environmental impact of the production of 1 kg of protein from dried BSF larvae fed with different proportions (5%, 10%, 20%, and 25%) of *Schizochytrium limacinum*.

Impact Category	Unit	5S	10S	20S	25S
Global warming	kg CO_2_ eq	1.6 × 10^5^ + 01	3.6 × 10^3^ + 01	5.4 × 10^5^ + 01	6.1 × 10 + 01
Freshwater eutrophication	kg P eq	7.6 × 10^9^ − 03	1.5 × 10^6^ − 02	2.3 × 10 − 02	2.5 × 10^5^ − 02
Land use	m^2^a crop eq	8.4 × 10^1^ − 01	1.7 × 10^5^ + 00	2.5 × 10^7^ + 00	2.8 × 10^6^ + 00
Mineral resource scarcity	kg Cu eq	5.5 × 10^9^ − 02	1.2 × 10^9^ − 01	1.9 × 10^6^ − 01	2.2 × 10 − 01
Fossil resource scarcity	kg oil eq	4.0 × 10^5^ + 00	9.0 × 10^9^ + 00	1.3 × 10^7^ + 01	1.5 × 10^4^ + 01
Water consumption	m^3^	3.6 × 10 − 01	7.9 × 10^5^ − 01	1.1 × 10^9^ + 00	1.3 × 10^3^ + 00

**Table 9:** Environmental impact of the production of 1 kg of protein from dried BSF larvae fed with different proportions (5%, 10%, 20%, and 25%) of *Isochrysis galbana*.

Impact Category	Unit	5I	10I	20I	25I
Global warming	kg CO_2_ eq	1.8 × 10^4^ + 01	2.7 × 10^6^ + 01	8.2 × 10 + 01	4.5 × 10^6^ + 01
Freshwater eutrophication	kg P eq	8.4 × 10^6^ − 03	1.2 × 10^1^ − 02	3.3 × 10^9^ − 02	1.9 × 10^1^ − 02
Land use	m^2^a crop eq	9.2 × 10^8^ − 01	1.3 × 10^4^ + 00	3.8 × 10^3^ + 00	2.1 × 10^5^ + 00
Mineral resource scarcity	kg Cu eq	6.2 × 10^6^ − 02	9.6 × 10^5^ − 02	2.9 × 10^8^ − 01	1.6 × 10^4^ − 01
Fossil resource scarcity	kg oil eq	4.5 × 10^2^ + 00	6.8 × 10^3^ + 00	2.0 × 10^7^ + 01	1.1 × 10^5^ + 01
Water consumption	m^3^	4.0 × 10^2^ − 01	6.0 × 10^2^ − 01	1.7 × 10^9^ + 00	9.9 × 10^7^ − 01

**Table 10:** Environmental impact of the production of 1 kg of lipid from dried BSF larvae fed with different proportions (5%, 10%, 20%, and 25%) of *Schizochytrium limacinum*.

Impact Category	Unit	5S	10S	20S	25S
Global warming	kg CO_2_ eq	6.0 × 10^6^ + 01	1.1 × 10^6^ + 02	1.1 × 10^9^ + 02	1.4 × 10^1^ + 02
Freshwater eutrophication	kg P eq	2.8 × 10^2^ − 02	4.9 × 10^9^ − 02	5.0 × 10 − 02	5.8 × 10^8^ − 02
Land use	m^2^a crop eq	3.0 × 10^9^ + 00	5.5 × 10^8^ + 00	5.6 × 10^1^ + 00	6.6 × 10^1^ + 00
Mineral resource scarcity	kg Cu eq	2.0 × 10^5^ − 01	4.1 × 10^3^ − 01	4.2 × 107 − 01	5.0 × 10^7^ − 01
Fossil resource scarcity	kg oil eq	1.4 × 10^9^ + 01	2.9 × 10^1^ + 01	2.9 × 10^8^ + 01	3.5 × 10^4^ + 01
Water consumption	m^3^	1.3 × 10^2^ + 00	2.5 × 10^4^ + 00	2,6 × 10 + 00	3.0 × 10^8^ + 00

**Table 11:** Environmental impact of the production of 1 kg of lipid from dried BSF larvae fed with different proportions (5%, 10%, 20%, and 25%) of *Isochrysis galbana*.

Impact Category	Unit	5I	10I	20I	25I
Global warming	kg CO_2_ eq	6.3 × 10^8^ + 01	1.0 × 10^8^ + 02	3.6 × 10^3^ + 02	1.6 × 10^8^ + 02
Freshwater eutrophication	kg P eq	2.9 × 10^4^ − 02	4.7 × 10^1^ − 02	1.5 × 10 − 01	7.0 × 10^5^ − 02
Land use	m^2^a crop eq	3.2 × 10^2^ + 00	5.2 × 10^3^ + 00	1.6 × 10^9^ + 01	7.9 × 10^6^ + 00
Mineral resource scarcity	kg Cu eq	2.1 × 10^7^ − 01	3.7 × 10^6^ − 01	1.3 × 10^2^ + 00	6.0 × 10^6^ − 01
Fossil resource scarcity	kg oil eq	1.5 × 10^7^ + 01	2.6 × 10^6^ + 01	9.1 × 10^5^ + 01	4.2 × 10^4^ + 01
Water consumption	m^3^	1.4 × 10 + 00	2.3 × 10^5^ + 00	7.9 × 10^4^ + 00	3.6 × 10^8^ + 00

## Data Availability

Data are contained within the article.
